# A Review on Metal-Organic Frameworks as Congenial Heterogeneous Catalysts for Potential Organic Transformations

**DOI:** 10.3389/fchem.2021.747615

**Published:** 2021-12-17

**Authors:** Kranthi Kumar Gangu, Sreekantha B. Jonnalagadda

**Affiliations:** ^1^ Vignan’s Institute of Information Technology, Visakhapatnam, India; ^2^ School of Chemistry and Physics, Westville Campus, University of KwaZulu-Natal, Durban, South Africa

**Keywords:** metal-organic frameworks (MOF), heterogeneous catalysis, reusability, catalytic active sites, green principles, value-added organic transformations

## Abstract

Metal-organic frameworks (MOFs) have emerged as versatile candidates of interest in heterogeneous catalysis. Recent research and developments with MOFs positively endorse their role as catalysts in generating invaluable organic compounds. To harness the full potential of MOFs in value-added organic transformation, a comprehensive look at how these materials are likely to involve in the catalytic processes is essential. Mainstays of MOFs such as metal nodes, linkers, encapsulation materials, and enveloped structures tend to produce capable catalytic active sites that offer solutions to reduce human efforts in developing new organic reactions. The main advantages of choosing MOFs as reusable catalysts are the flexible and robust skeleton, regular porosity, high pore volume, and accessible synthesis accompanied with cost-effectiveness. As hosts for active metals, sole MOFs, modified MOFs, and MOFs have made remarkable advances as solid catalysts. The extensive exploration of the MOFs possibly led to their fast adoption in fabricating new biological molecules such as pyridines, quinolines, quinazolinones, imines, and their derivatives. This review covers the varied MOFs and their catalytic properties in facilitating the selective formation of the product organic moieties and interprets MOF’s property responsible for their elegant performance.

## Introduction

Eco-friendly practices to overcome the concerns related to organic transformations is crucial. The excess use of toxic catalysts cause health hazards and result in the imbalance of ecology. Globally, several catalytic management practices using hazardous chemicals as catalysts have been rejected to safeguard the environment (Dhakshinamoorthy et al., 2011; Sun et al., 2016; Zhao et al., 2016). Many such chemicals as catalysts have already shown the tendency to accumulate and magnify the reaction host system. Thus, these days, focus on environmentally benign catalysts is emerging on a larger scale to counter the catalyst hazards effectively. Heterogeneous catalysis is another promising approach for detoxifying organic reaction contamination caused by harmful chemicals as catalysts (Wolfe et al., 1999; Sun et al., 2014; Kou et al., 2017). With the development of state-of-the-art technology, a roadmap towards cleaner catalytic practices can be envisaged. Difficulties with isolating, reusing, and thermal instability with homogeneous catalysts in organic transformation have significantly encouraged heterogeneous catalysts. The advance with metal-organic frameworks (MOFs) is a step closer to the reality of their use as solid catalysts in industrial applications.

The MOFs comprise metal ions/clusters (inorganic nodes) and organic linkers, enabling them to be a promising class of materials (Kitagawa et al., 2004; Furukawa et al., 2013; Gangu et al., 2017a; He et al., 2018). MOFs are porous coordination polymers with unique tunable pore structure, diverse composition, and versatile functionality with high porosity. The flexibilities in the design and unique properties are crucial to the adoption of MOFs in heterogeneous catalysis. The primary limitation of heterogeneous catalysts is the difficulty in their functionalization and mass transport. MOFs can alleviate such constraints and create a new vista in the value-added organic transformations (Lee et al., 2009; Lu et al., 2018; Woellner et al., 2018). The probable advantages of the MOFs as heterogeneous catalysts are their coordinatively unsaturated sites, high density of transition metals, pore size/surface area, size selectivity, activation of oxidant by Lewis acid, and increased stability. These characteristics and the substrate activation by the Lewis acid has considerably enhanced the acceptance of MOFs as catalysts in modern organic synthesis (Farrusseng et al., 2009; Gangu et al., 2016). Solid catalysts shorten the reaction time and almost minimize the use of hazardous chemicals. For example, the preparation of 4-methylthiazole (4-MT), an essential intermediate in synthesizing fungicides, involves a five-step process. Employing a solid base, namely, the Cs-containing zeolite, as a catalyst reduces the five-step to two-step process and avoids using hazardous chlorine and carbon disulfide in the reaction process (Davis, 1993). After that, many solid catalysts were reported, and catalytic species were introduced to porous supports like MOFs to overcome the reaction hurdles.

**SCHEME 1:**
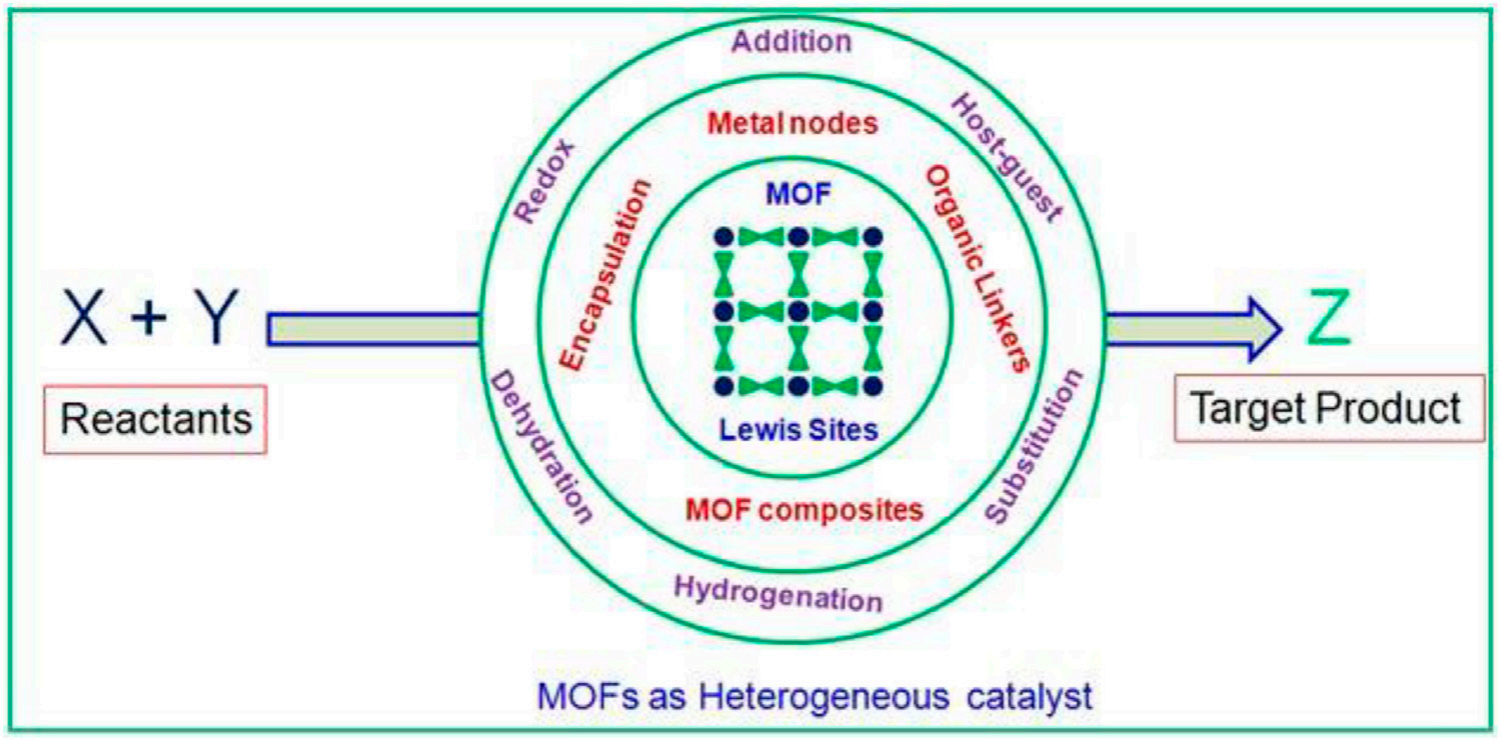
Perspective view of review enlightens the organic transformation with MOFs as solid heterogeneous catalysts.

MOFs are crystalline materials with high specific surface area up to 10,400 m^2^g^−1^, low density (0.13 g cm^−3^), and exemplary pore aperture (98 Å) with multi-topic bridging linkers transforming into extensive porous networks benefitting to heterogeneous catalysis (Wang and Cohen, 2009; Corma et al., 2010; Hu and Zhao, 2017; Zhao et al., 2018a). The chemical environment of cages/channels in the MOFs is also vital in the efficiency of catalysis. The pore sizes between the ranges of 20–500 Å are presumably allowed the large guest species or functional materials to wrap in the framework structure leading to the enhanced catalytic activity (Rowsell and Yaghi, 2004; Bosch et al., 2017; Burtch et al., 2018). In many cases, the catalytic activity of MOFs can be elicited many ways, like metal ions themselves act as active catalytic sites, linker/mixed linkers as active sites. Impregnating functional materials like metal nanoparticles, polyoxometalates, enzymes, quantum dots, silica, molecular species, and polymers to improve MOFs’ catalytic characteristics is a suitable way (Hattori, 1995; Valvekens et al., 2014). In the case of metal ions as active catalytic sites, unsaturated coordination sites are available on the surfaces of MOFs even though metal sites are completely ligated with strong organic substrates. The removal of volatile solvents like water, alcohol, DMF and acetonitriles, etc., prompts the catalytic action of metal nodes due to the creation of unsaturated metal coordination sites (Roeser et al., 2012; Yuan et al., 2018). Design and construction of MOFs for rational assembly of their framework components for acclimatization to heterogeneous catalysis, many attempts have been made to get desired MOF catalyst. Post-modification, building with metallosalens, metalloporphyrins, amino acid moieties are helpful to create a multitude of MOF catalysts (Li et al., 1999; Xu et al., 2019). The advantage of MOFs over other porous materials like zeolites is the persistence of microporosity after solvent removal and thermal stability, placing MOFs at the front line with enzymes, activated carbons and zeolites. Based on the type of active catalytic sites of MOFs, they can be employed in various organic reactions. The Lewis-acid sites catalyze cyanosilylation of acetone or benzaldehyde, and the Lewis basic sites facilitate the Knoevenagel condensation of benzaldehyde (Wang et al., 2013; Dang et al., 2016). Marking all these remarkable propensities, various organic transformations like aldol condensation, oxidation reactions, Knoevenagel condensation, Friedel–Crafts reactions, Suzuki coupling, etc., have been explored with MOFs as catalyst supports or solid catalysts (Scheme 1) (Sholl and Lively, 2015; Huang et al., 2016; Jiao et al., 2018; Pham et al., 2018).

**FIGURE 1:**
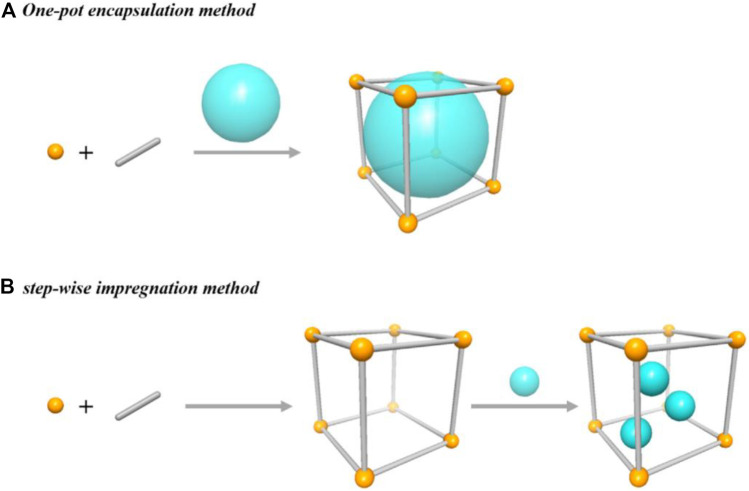
Cartoon diagram of two approaches for the integration of catalytic species into MOF pores. [Reproduced from Ref. (Gong et al., 2020)].

Breaking and making chemical bonds is an essential process in the conversion of reactants into valuable products. MOF’s role in the catalysis makes the task of difficult cleavage of old bonds in the reactants facile and facilitates the formation of new bonds for the target products. MOFs as catalysts enhance atom efficiency and turnover number (TON) in organic synthesis. MOFs offer intrinsic selectivity and activity with modified catalytic nature. The catalytic properties of the metals (alkaline Earth and hybrid metal nodes) and ligands (N-containing ligands and structural phenolates) in MOFs can be further enriched through the functionalization of metal sites (grafting of diamines) and ligands (introduction of amines and introduction of metal ions) (Huang et al., 2017; Islamoglu et al., 2017; Kang et al., 2019; Wen et al., 2018; Pascanu et al., 2016). Incorporating acidic/basic guest catalytic sites into the MOFs is feasible without altering their indispensable topological structure. Impregnation and encapsulation of guest species within the pores are achievable by two approaches. One-pot encapsulation and step-wise impregnation, as shown in Figure 1. In both cases, the diameter of guest species and pore aperture of MOF plays a vital role in engineering guest molecules into the pores (Zhao, 2018; Gong et al., 2020).

On the other hand, MOF composites are practical approaches to enhance catalytic activity against the pristine MOFs employed in the reaction (Poonam and Rajendra, 2017; Zhai et al., 2019; Gangu et al., 2019). Due to fewer active sites and meagre thermal and mechanical properties, a lower catalytic activity restricted the bare MOFs as catalysts. MOF composites/hybrids synergistic effect is the remarkable feature to deal with the shortcomings of individual components of the composite. Integrating MOFs with functional materials can be surmounted through two possible ways. In one approach, MOF acts as a support to accommodate active materials like metal nanoparticles. In the second case, functional materials like silica and polymers support MOFs for enhancing their chemical and mechanic strength (Juan-Alcañiz et al., 2012; Gangu et al., 2017b). In the former case, it’s possible to mitigate the aggregation and leaching of functional materials, whereas the latter facilitates the congenial catalytic formulation for superb catalytic applications.

## Design Strategies of MOF Composites/Hybrids

**FIGURE 2:**
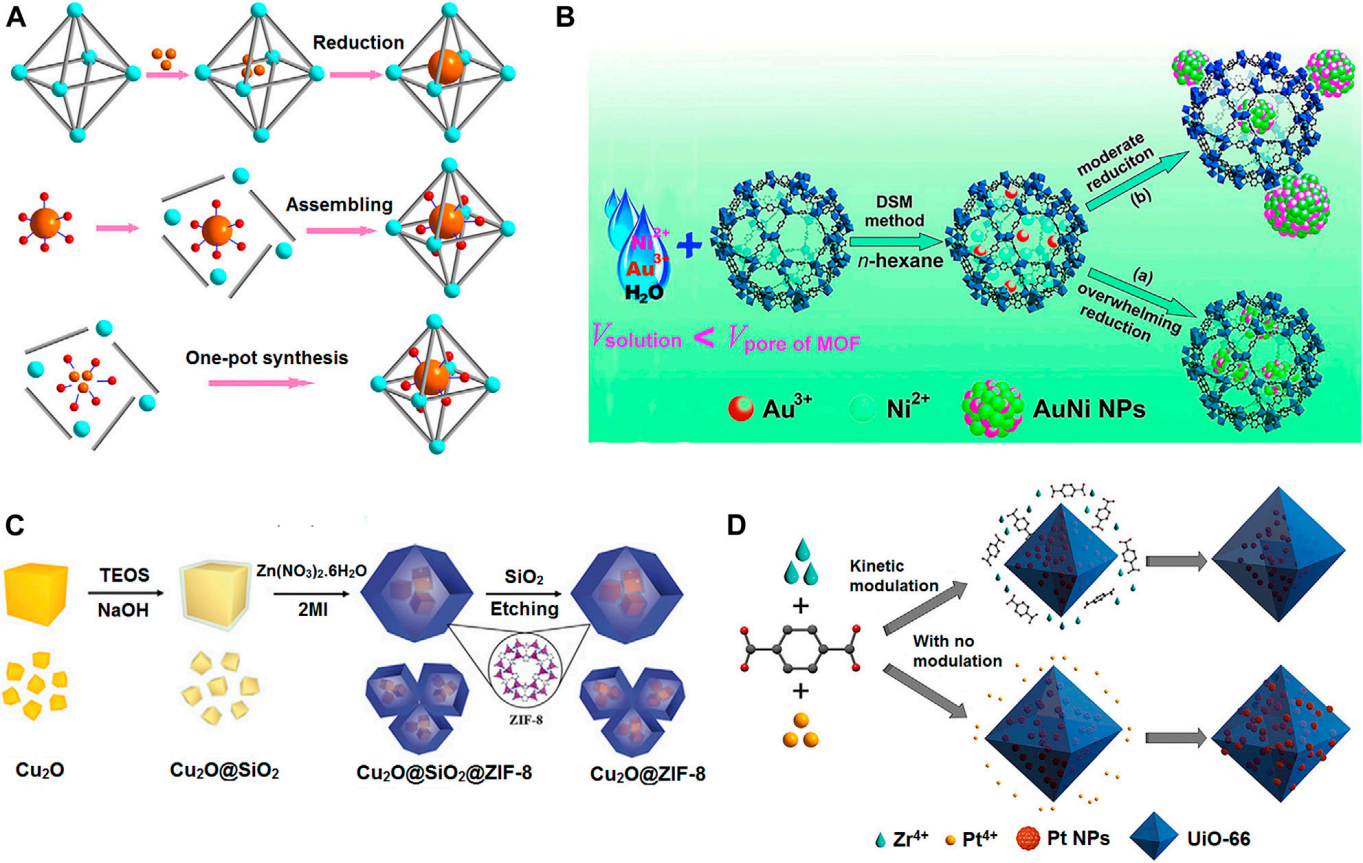
Different available strategies for the fabrication of MOF composites/hybrids. **(A)** Strategies for fabrication of MOF-metal NP composites including ship-in-bottle, bottle-around-ship, and one-pot synthesis. Cyan, inorganic nodes of MOFs; gray, organic linkers; orange, metal precursors or NPs; red, stabilizing agents. **(B)** Schematic representation of immobilization of the AuNi nanoparticles by an MIL-101 matrix using DSM combined with a liquid-phase CCR strategy. **(C) **Schematic illustration for the synthesis of the Cu2O@ZIF-8 composite. **(D)** Incorporation of Pt NPs in a UiO-66 MOF by means of an *in situ* one-step protocol with kinetic modulation by H2/acetic acid (top) and with no modulation (bottom). [Reproduced from Ref. (Chen and Xu, 2019)].

MOF composites/hybrids are systematically designable through various routes. Ship-in-bottle, bottle-around-ship, and one-pot synthesis are promising techniques to fabricate MOF hybrids, as shown in Figure 2A. In another strategy, metal nanoparticles can be encapsulated into MOF matrix using liquid-phase concentration-controlled reduction (CCR) strategy as shown in Figure 2B. In other words, surfactant-assisted MOF/metal nanoparticle composites is an excellent route to acquire MOF shell around metal nanoparticles. The surfactant is used to elude the aggregation of nanoparticles and the self-nucleation process of MOFs. The surfactant removal from the surfaces of MOF/metal nanoparticles surface is the associated problem. The hard-template method avoiding that limitation uses inert silica or active metal oxides as templates to synthesize MOF-encapsulated metal nanoparticles as in Figure 2C with a free capping agent-surface. As in Figure 2D, the one-pot technique of direct loading of metal nanoparticles to MOF has received good attention as it reduces production time and cost and is easy for scale-up. In this technique, assembly of MOFs around metal nanoparticles with modulators and solvent can be achievable. The nucleation/growth of metal nanoparticles and MOF are crucial to obtain appropriate MOF hybrid materials (Zhu and Xu, 2014; Chen and Wu, 2018; Chen and Xu, 2019).

## MOFs/Modified MOFs as Solid Catalysts in the Organic Transformations

Many researchers have been conducting exemplary trials using MOFs as solid catalysts for fiscal benefits and a sustainable environment in the fine chemical and medicinal drug industries due to increasing production costs and cumbersome synthetic routes. The following discussion enlightens the critical contributions of various MOFs and their role in the facile organic synthesis (Table 1).

**TABLE 1:** Different MOFs and their composites as heterogeneous catalysts in organic transformations discussed in this review.

MOF/Solid catalyst	References number	Catalytic activity	Organic reaction
[(Cu(L)_2_·(H_2_O)_2_·(NO3)_2_]_n_ (1) Where L = 4-(5-methyl-3-pyridine)-1,2,4-triazole	Guo (2019)	Cu (II) metal sites- Lewis acid catalysis	Conversion of CO_2_ into cyclic carbonates
Pd@Cu-BDC/Py-SI (2)	Rostamnia et al. (2016)	The Schiff base Pd complex molecules on the cage of Pd@Cu-BDC/Py-SI	Production of biaryls over the Suzuki reaction
[Cu_2_(CN)_2_(BPY)] (3) CuCl_2_ as a metal source,4,4′-bipyridine (BPY) as bridged ligand	Shi et al. (2020)	Cu(I) sites in the MOF facilitate catalysis	cyclization of tertiary propargylic alcohols with CO_2_
Au@Cu(II)-MOF (4) Cu(OAc)_2_ and pyridyl substituted diketonate ligand	Wang et al. (2016)	Cu(II)-the framework is a valuable platform for supporting and stabilizing Au NPs	Knoevenagel condensation and benzyl alcohol oxidation
Cd (II)-MOF (5) 1,2-diphenylethane-1,2-dionebisisonicotinylhydrazone (H_2_DDIH) as dihydrazone linker	Tom and Kurup (2021)	water coordinated Cd(II) ions act as active Lewis acidic catalytic sites	Knoevenagel reaction
MOF-199 (6) copper nitrate and 1,3,5‐benzenetricarboxylic acid	Raut et al. (2021)	Cu(II) catalytic active sites	Preparation of 2,2,4‐trimethyl‐1,2‐dihydroquinolines reacting different anilines and ketones
[Fe_3_(BTC) (EDB)_2_12.27H_2_O] (7) BTC = 1,3,5-benzenetricarboxylate and EDB^2-^ = 4,4′-ethynylenedibenzoate	Nakanishi and Bolm (2007); To et al. (2019)	The iron catalyzed decarboxylation of phenylacetic acid is occurred promptly	Preparatio of 2-phenylquinazolin-4(3H)-one from Phenylacetic acid and 2-aminobenzamide
Cu^2+^ captured into UiO-66–(COOH)_2_ (8) (Cu@UiO-1, Cu@UiO-2 and Cu@UiO-3)	Zhao et al. (2018b)	Copper catalytic sites enables the catalytic reaction in Cu@UiO-1 more cheaply	olefin epoxidation
UiO-66(Ce) (9)	Nagarjun et al. (2021)	Ce^4+^ ions as active sites in UiO-66(Ce)	Aerobic oxidation of benzyl amines
Ni@MIL-125(Ti)-NH_2_ (10)	Chen et al. (2020)	Schiff base nickel (II) complex acts as active catalytic site	ethylene oligomerization
L-proline grafted [Zr_6_O_6_(OH)_2_ (tdc)_4_(CH_3_COO)_2_], DUT-67-pro (11)	Nguyen et al. (2018)	L-proline chiral catalytic sites	cyclohexanone to trans-β-nitrostyrene
IRMOF-8 (12) Zinc nitrate tetrahydrate and 2,6-naphthalenedicarboxylic acid	Nguyen et al. (2011)	Lewis acidic sites	Friedel–Crafts acylation
Adenine functionalized Mn-MOF-74 (13)	Feng et al. (2020)	Lewis acidic-basic active sites	Synthesis of cyclic carbonates
[Al (OH) (hfipbb)] (14), AlPF-1 [In (O_2_C_2_H_4_)_0.5_ (hfipbb)] (15),InPF-11β and [Ga (OH) (hfipbb)] [16], GaPF-1	Aguirre-Díaz et al. (2015)	Lewis acid and base sites in the catalyst	Strecker reaction
MIL-101 (17)	Sun and Gao (2020)	sulfonic and Cr(III) sites collaboratively to enhance the activity	oxidation of cyclohexene

Cu (II) based MOFs have been prepared in numerous laboratories to route the simplified and miniature technology in organic synthesis. Guo (2019) fabricated Cu(II) MOF, namely [(Cu(L)_2_·(H_2_O)_2_·(NO3)_2_]_n_ (**1**) using 4-(5-methyl-3-pyridine)-1,2,4-triazole (namely L) as an assembled ligand in the MOF 2D layer structure. Instead of carboxylic acid groups in the organic linker, pyridine rings were adopted to build cationic MOFs, which showed good behaviour to act as host materials for capturing pollutants. Enhanced atmospheric carbon dioxide levels cause severe problems, and its capturing and transformation are crucial for the environment and climate. The CO_2_ conversion into industrially viable cyclic carbonates through reaction with epoxides requires an effective catalyst for easy conversion. Available Cu (II) metal sites in this MOF suitable for Lewis acid catalysis prompt the development of interactions between CO_2_ and the oxygen atom of epoxide reactant. In this reaction, tetrabutylammonium bromide (TBABr) is also employed as a co-catalyst in the easy process to form final cyclic carbonates. On the other hand, the Suzuki-Miyaura coupling reaction is a pivotal organic response, and it is carried out mainly in Pd-containing catalysts. The leaching of expensive metal and its toxicity demands an alternative catalytic system to the Pd catalytic substances. MOFs facilitate the immobilization of the Pd on MOF’s structure for designing the appropriate heterogeneous system for the Suzuki-Miyaura coupling reaction. Rostamnia et al. (2016) for grafting Pd ions, Cu-BDC MOF was constructed using copper nitrate and terephthalic acid (BDC) as precursors. In the subsequent step, Cu-BDC was activated with pyridyl-salicylimine (Py-SI) moiety, and afterwards, Pd precursor, i.e. PdCl_2,_ was added to get the final heterogeneous catalyst, Pd@Cu-BDC/Py-SI (**2**). After assigning optimizing conditions, **2** produced the target product biaryls over the Suzuki reaction. Atomic absorption spectroscopy studies revealed no Pd ions detected from the catalyst system in the reaction solution until the fourth recycled run. The Schiff base Pd complex molecules on the cage of Cu-BDC MOF produced Suzuki-coupling product, biaryls in high yields without any other side-products. In another study, the exclusive library of excellent functions of cyanides, particularly cyano-bridged coordination polymers in the heterogeneous catalysis are taken into consideration and developed a cyano-based MOF, namely [Cu_2_(CN)_2_(BPY)] (**3**) with the incorporation of precursors such as CuCl_2_ as a metal source,4,4′-bipyridine (BPY) as bridged ligand and Na_4_W_10_O_32_ in a mixed solvent system of H_2_O and CH_3_CN. This study used acetonitrile instead of conventionally used toxic enabled inorganic cyanides and HCN. Synergistic effect of Cu^2+^ ions and W_10_O_32_^4-^ under synthetic hydrothermal conditions, C–CN bond of acetonitrile was cleaved and produced CN^−^ion. The catalytic efficacy of **3** for carboxylic cyclization of propargylic alcohols was reported (Shi et al., 2020). Cu (I) sites in the complex facilitate catalyzed cyclization of tertiary propargylic alcohols with CO_2_. In another study, Luz et al. (2012) have reported the Cu-MOF, [Cu(BDC)] as a solid catalyst for a three-component coupling reaction. Amine, aldehyde and alkyne are three precursors that undergo reaction and produce propargyl amines, prime synthetic intermediates in synthesizing therapeutic drugs and natural product molecules. In this study, several Cu based MOFs were prepared and tested for their heterogeneous catalytic behaviour. The observation that even if having probable copper active sites in series of Cu-MOF, copper terephthalate [Cu(BDC)] MOF showed best results with high conversion percentage. The reason attributed that poisoning of the catalyst occurs with reverse blocking of pores. Thus, the structure orientation and allied properties of designated MOF play a significant role in heterogeneous catalysis.

**FIGURE 3:**
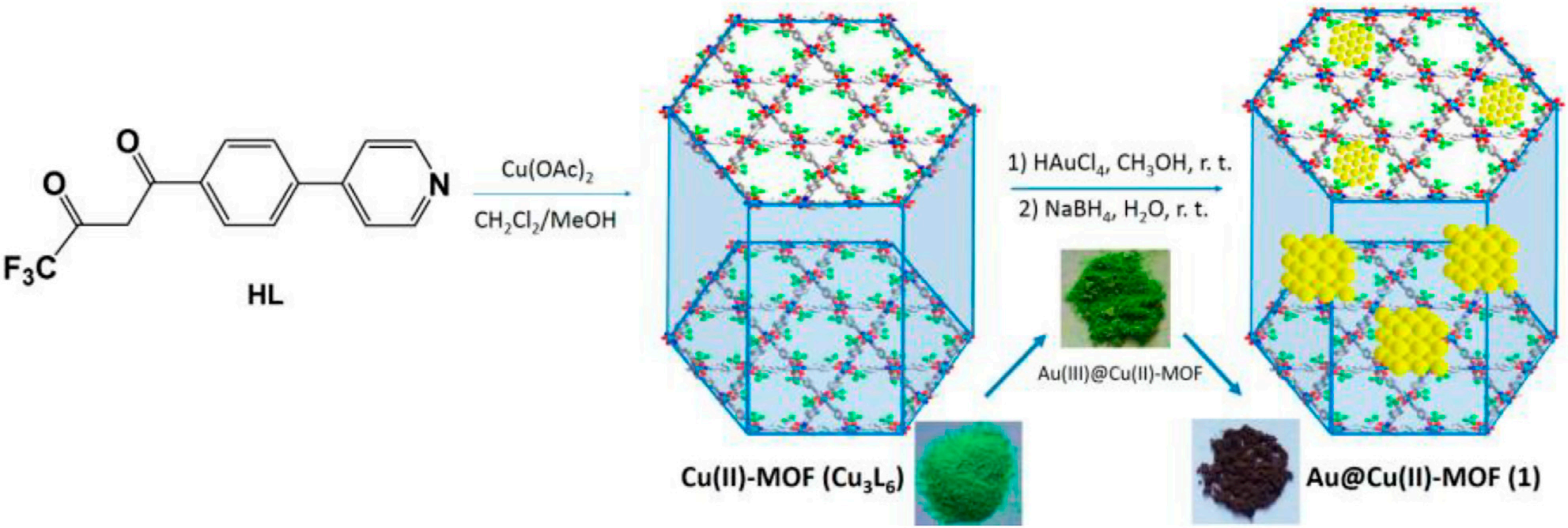
Schematic representation of the synthesis of Cu (II)-MOF and HAuCl_4_ [Reproduced from Ref. (Wang et al., 2016)].

Wang et al. (2016) have used the porous behaviour of MOFs as support material for uploading active gold nanoparticles (Au NPs) for implementing catalytic performance in the many organic reactions. The study revealed that even if MOFs possess competent organic-inorganic composition for catalytic activity, the incorporation of metal NPs desirably enhances the catalytic activity and would lead to novel catalytic characteristics compared to their new counterparts. The author has designed Au@Cu(II)-MOF (**4**) (Figure 3) for Knoevenagel condensation and benzyl alcohol oxidation. Cu(II)-MOF was prepared by the combination of Cu(OAc)_2_ and pyridyl substituted diketonate ligand and later added to the methanol solution of chloroauric acid for the final solid catalyst **4**.

The aerobic oxidation of benzyl alcohol to valuable benzaldehyde in the presence of Au@Cu(II)-MOF under optimized conditions gave excellent conversion of about 98% whereas conversion only 3% with Au NPs free catalyst, which demonstrates the requirement of Au NPs in the Cu(II)-MOF. Overall Cu(II)-the framework is a valuable platform in this study for supporting and stabilizing Au NPs.

The fundamental strategy in designing MOFs with specialized catalytic properties is selecting appropriate ligands with suitable functional groups. Polytopic ligands compared to ditopic ligands accommodated with multiple catalytic centres for heterogeneous catalysis. Tom et al. (Tom and Kurup, 2021) chose double hydrazone ligands, favouring the novel architectural patterns in preference to rigid ligands like dicarboxylates and bipyridines. Different unique orientations of nitrogen atoms and zigzag conformation of–CR = N–N=CR–moiety in the structure result in ligand binding in exodentate fashion to metal nodes. 1,2-diphenylethane-1,2-dione bisisonicotinylhydrazone (H_2_DDIH) as dihydrazone linker formed the MOF with Cd(II) metal ions. In the Cd (II)-MOF [**5**] structure, labile water coordinated Cd(II) ions act as active Lewis acidic catalytic sites, which exhibited Knoevenagel reaction with 96% conversion in 15 min reaction time at room temperature. No traces of Cd^2+^ species were noticed during the process under optimized conditions. These leaching experiments and thermal stability have shown the material’s viability in heterogeneous catalysis and can be an excellent alternative to other solid catalysts for the Knoevenagel condensation.

Raut et al. (2021) have developed MOF-199 (**6**) using the copper nitrate and 1,3,5‐benzenetricarboxylic acid and explored it as a catalyst for preparing 2,2,4‐trimethyl‐1,2‐dihydroquinolines reacting different anilines and ketones. The Quinoline ring is a multi bio-active moiety that belongs to the quinine group involved in various drug syntheses and the well-known antimalarial drug. The classical quinoline synthesis is through Skraup synthesis, which has some drawbacks (Radhakrishna et al., 2017; Basavarajaiah and Nagesh, 2021). MOF-199 has been used as an efficient, recyclable catalyst in esterification, Ullmann‐type coupling, and oxidative dehydrogenation reactions. It also worked efficiently in the synthesis of quinoline derivatives. The appropriate selection of copper salts in the **6** construction is also significant because no desired product development was seen with other copper salts except copper nitrate, which indicated that selecting the type of metal salts in the designing of MOF’s is crucial. In the synthesis of quinolones, excellent conversions were reported with 2.5 mol% MOF-199 without any solvent or reagents. In another study, biological active nitrogen-containing quinazolinones were synthesized in the presence of mixed-linker iron-based MOF [Fe_3_(BTC) (EDB)_2_12.27H_2_O] (**7**) as a catalyst, where BTC = 1,3,5-benzenetricarboxylate and EDB^2-^ = 4,4′-ethynylenedibenzoate. Phenylacetic acid and 2-aminobenzamide underwent a one-pot chemical reaction to form 2-phenylquinazolin-4(3H)-one in the presence of iron-based MOF produced excellent yields compared to other solid catalysts like Fe_3_O(BDC)_3_, Fe_3_O(BPDC)_3_, Cu_2_(OBA)_2_(BPY), Cu-MOF-199 and Co-ZIF-67 (Nakanishi and Bolm, 2007; To et al., 2019). The iron-catalyzed decarboxylation of phenylacetic acid through SP^3^hybridised carbon and hydrogen bond activation, accelarates the cyclization of intermediate with 2-aminobenzamides to produce the target product, quinazolinone. The recyclability of catalysts without hampering catalytic performance is helpful in the valuable organic transformations and chemical industry.

Pyridine containing heterocycles exhibit exciting biological activities. For example, 2-amino-6-(arylthio)pyridine-3,5-dicarbonitrile scaffolds are widely used to make various pharmaceutical agents treat the adenosine receptors caused by Parkinson’s, hypoxia, epilepsy, cancer, asthma, and cardiovascular diseases (Chang et al., 2005; de Los Ríos et al., 2010). Pyridines products are active inhibitors of adenosine receptors, and thus the synthesis of these molecules in a feasible way is also furthering advances in disease treatments. In this connection, to develop a practical environmentally benign synthetic route, Thimmaiah et al. (2012) have identified two isostructural Cd (II) and Zn (II) based MOFs as solid catalysts to synthesize pyridine compounds. Benzaldehyde, malononitrile, and thiophenol underwent a one-pot reaction facilitated by Cd and Zn(II) MOFs producing the 2-amino-6-(arylthio) pyridine-3,5-dicarbonitrile. In the solvent-free conditions, both Cd(II) and Zn(II) MOFs facilitated the final product in 30 min, with 87 and 86% yields, respectively. The MOF-mediated green synthesis of pyridine derivative with easy operation realized the desired transformation.

**FIGURE 4:**
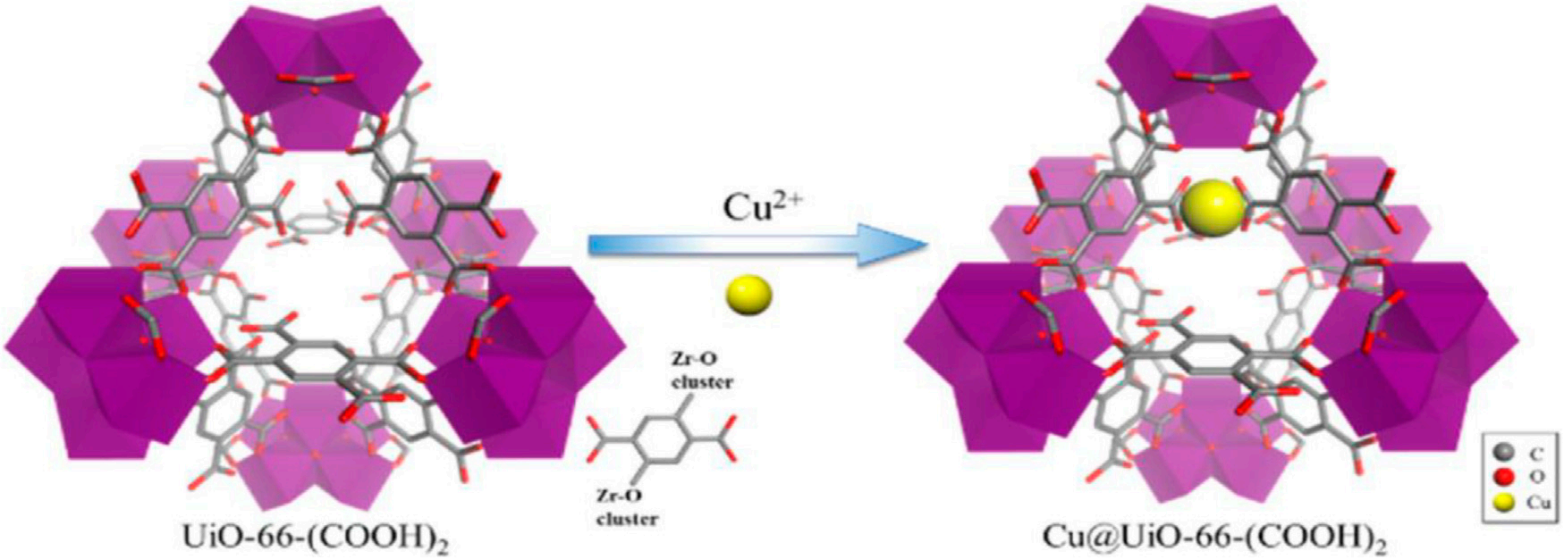
Zr-based MOF Using Cu^2+^ ions [Reproduced form Ref. (Zhao et al., 2018b)].

Zhao et al. (2018b) have modified Zr-based MOF UiO-66-(COOH)_2_ (**8**) with three different copper salts, generating Cu@UiO-1, Cu@UiO-2 and Cu@UiO-3 materials, and employed in the olefin epoxidation as catalysts. Microporous nature and ability to incorporate other chemical functionalities to design more transition metal-based catalysts with developed MOFs through post-synthetic modification. In this way, abundant, inexpensive and non-toxic Cu^2+^ captured into UiO-66–(COOH)_2_ MOF post synthetically in this study as shown in Figure 4.

Different copper salts like Cu(NO_3_)_2_·3H_2_O for Cu@UiO-1, CuCl_2_·2H_2_O for Cu@UiO-2 and CuSO_4_·5H_2_O for Cu@UiO-3 were used. The powder XRD patterns confirmed no destruction of a crystalline structure after post-modification of UiO-66-(COOH)_2_ with copper ions. Although the cyclooctene epoxidation was achieved successfully with all three catalysts, Cu@UiO-1 showed superior catalytic performance (>99% conversion). The study showed that epoxidation of olefins could be carried out with Cu@UiO-1 cheaply instead of using expensive titanium (IV), molybdenum (VI), and manganese (II) metal ions. In another study, post-synthetic modification (PSM) of a MOF is expected to have much more recognition and received several exciting catalytic properties. PSM is a tunable, modular and promising approach for discovering active, robust and selective MOF catalysts for making the process of drug innovations from the lab to market easier (Sha and Chuan-De, 2014; Chughtai et al., 2015). The careful selection of metal ion and organic ligands produce MOF structures with favourable attributes like unsaturated metal sites, specific pore and chiral topologies with PSM. Analogous MOFs with UiO-66 frameworks were synthesized with different transition metal ions like Ti (IV), Hf (IV), Th (IV), U (IV), and Ce(IV) possessed high thermal and chemical stabilities. In this connection, Nagarjun et al. (2021) have reported a UiO-66 (Ce) (**9**) metal-organic framework for the aerobic oxidation of benzyl amines. Synthesis of imines from amines conventionally requires catalyst and oxidant, but the separation of oxidant from the reaction mixture is tedious and not environmentally benign. Coordinatively unsaturated metal sites behaving as Lewis acid sites, particularly in metal nanoparticles encapsulated MOFs are responsible for encouraging oxidation of various substrates (Li et al., 2017; Amarajothi et al., 2020). Active sites in **9** were identified as Ce^4+^ ions, and it was confirmed with n-CeO_2_ used as a catalyst. With n-CeO_2_, Ce^4+^ promoted the reaction forward, but activity was three-fold lower than with **9**. The reason attributed to the coordination environment and availability of a high number of active sites in **9** probably indicates the superior activity of MOF. The study suggests that the coordination structure, possible active sites, surface area and oxophilicity supported the potential reaction pathway in the aerobic oxidation of amines.

Tanabe et al. (Tanabe and Cohen, 2010) have modified parent MOF (UMCM-1-NH_2_) with different metal ions and chelating groups. The results showed that four modified MOFs resemble the structure and thermal stabilities but displayed various catalytic properties in the epoxide ring-opening reactions. The reason attributed is that during PSM within the framework structure well defined single-site catalysts were achieved. This altered with combinations of ligand and metal ions employed in the PSM and MOF environment.

Friedel–Crafts alkylation is another significant organic reaction that is particularly important to the chemical and petroleum industries. The traditional AlCl_3_, ZnCl_2_ and FeCl_3_, catalyzed systems are highly moisture-sensitive, demanding alternate catalysts that are efficient, safe, and environment-friendly. MOFs in general and MOF-5 in particular address the issues efficiently (Opelt et al., 2008; Dang et al., 2017). Highly porous MOF-5 reported as efficient solid catalyst for liquid-phase Friedel–Crafts alkylation reactions. In a study by Phan et al. (2010) MOF-5 showed efficient heterogeneity and was reused without any significant degradation in the catalytic performance. Lewis acid sites of MOF-5 exhibited feasible alkylation of benzyl halide and toluene, but it was inactive towards alkylation of toluene and benzyl alcohol, demonstrating selectivity.

**FIGURE 5:**
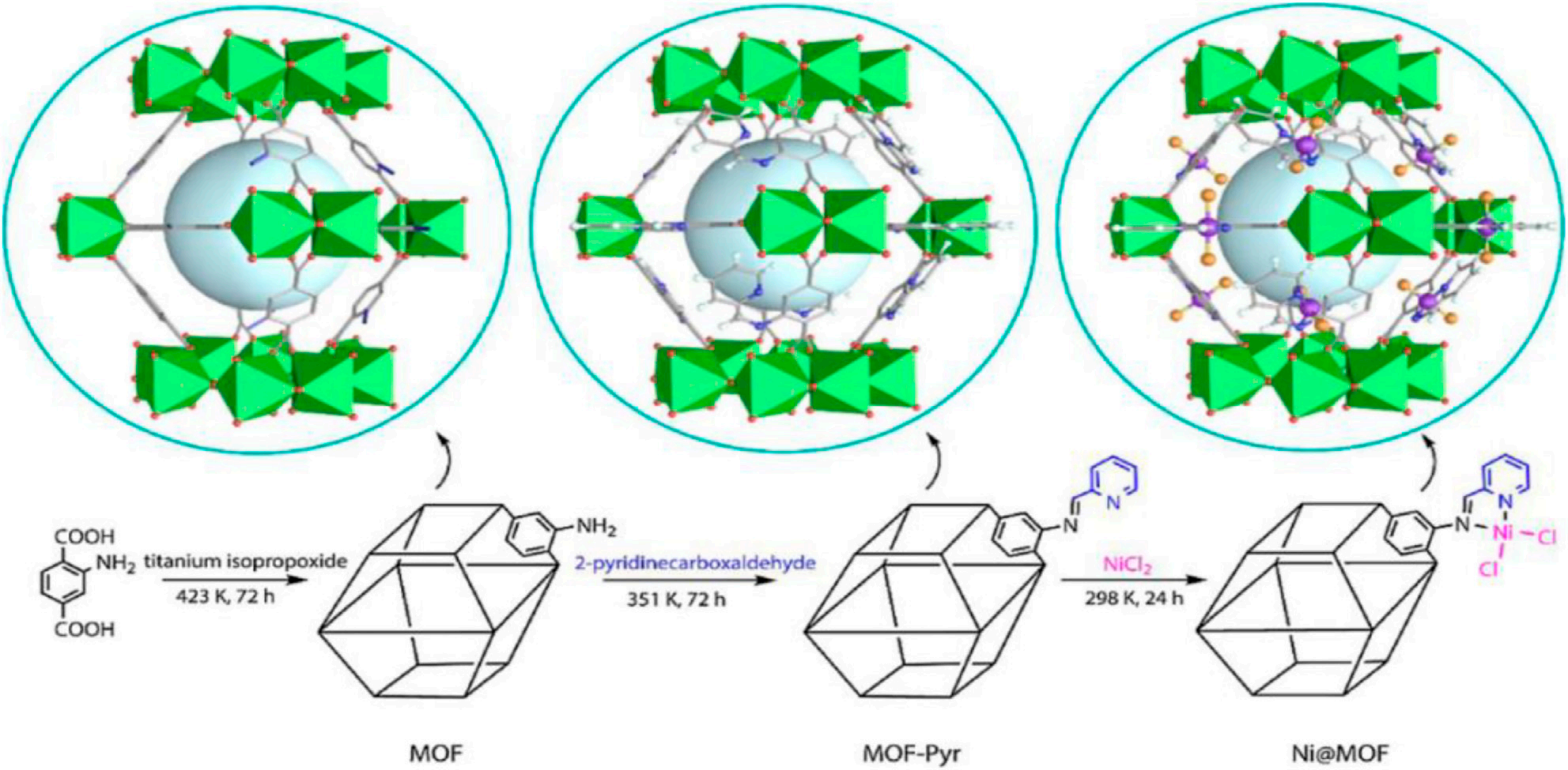
Synthetic routes of Ni@MOF [Reproduced from Ref. (Chen et al., 2020)].

Linear α-olefins (LAOs) prepare many detergents, oil field chemicals, lubricants and plasticizers. Ethylene oligomerization is one of the most crucial chemical processes to obtain LAOs. Alkylaluminium and late-transition metal catalysts are used chiefly as catalytic substances in ethylene oligomerization. Nickel-based catalysts are among the topmost catalysts in ethylene oligomerization, but their homogenous nature lowers broader utilization (Finiels et al., 2014; Arrozi et al., 2019). Addressing the problem, Chen et al. (2020) developed Ni@MOF as a heterogeneous catalyst for ethylene oligomerization. Ni@MOF was prepared by the post-synthetic modification of MIL-125(Ti)-NH_2_ (**10**), and treated with pyridine and terephthalate amino groups, followed by metalation with nickel (Figure 5). Ethylene oligomerization does not proceed without nickel, indicating Ti could not be the active metal site in this MOF catalyst. Schiff base nickel (II) complex grafted over the MOF structure reliably perfect pore system for the production of oligomers.

Nguyen et al. (2018) have reported post synthetically modified Zr-MOF (DUT-67) with chiral molecules for C-C bond formation. Small chiral amines like L-proline have been efficiently used as organocatalysts in Michael addition of ketones to nitroalkenes. To provide heterogeneity, L-proline is anchored onto insoluble DUT-67 as it is chemically and thermally more stable. DUT-67 is an 8-connected cluster of {[Zr_6_ (μ_3_-O)_6_ (μ_3_-OH)_2_]^10+^} and this cluster grafted with L-proline chiral catalytic sites, resulting in DUT-67-pro (**11**). Even if grating L-proline, no changes in the crystalline structure and powder XRD pattern match the parent DUT-67. Catalytic studies revealed that DUT-67-pro showed good performance in the C-C bond formation of cyclohexanone to trans-β-nitrostyrene with a 96% yield than the homogeneous L-proline. But, L-proline catalytic sites are responsible for the catalytic reactions rather than the basic MOF structure. Here, DUT-67 acted as good solid support and to gain excellent heterogeneity character.

Friedel–Crafts acylation is another significant organic reaction with acid chlorides for various applications in industrial chemistry. Traditionally, strong Lewis acids such as AlCl_3_, SnCl_4_, TiCl_3_, FeCl_3_ have been employed in the reaction mixture as catalysts. Drawbacks such as high amount requirement, corrosive nature, complex purification procedures and toxicity problems forced the search for alternative catalysts. MOF like IRMOF-8 alleviate such issues and produce an optimal yield of the targeted aromatic ketones. The employment of IRMOF-8 (**12**) as a solid catalyst is advantageous for maintaining anhydrous conditions. Generally, Lewis acids are moisture sensitive, and demand keeps moisture-free requirements to execute the reaction. Zinc nitrate tetrahydrate and 2,6-naphthalenedicarboxylic acid are precursors for the **12**, which possesses high porous nature. As per the study conducted by Nguyen et al., (Nguyen et al., 2011) toluene and benzoyl chloride in the Friedel–Crafts acylation reaction proceeded with IRMOF-8 catalytic amount of 1–5 mol%. MOF-5 was also examined in this study, but slightly better results were obtained with IRMOF-8 and suggested using MOF-5 as an alternative catalyst for the Friedel–Crafts acylation reaction.

**FIGURE 6:**
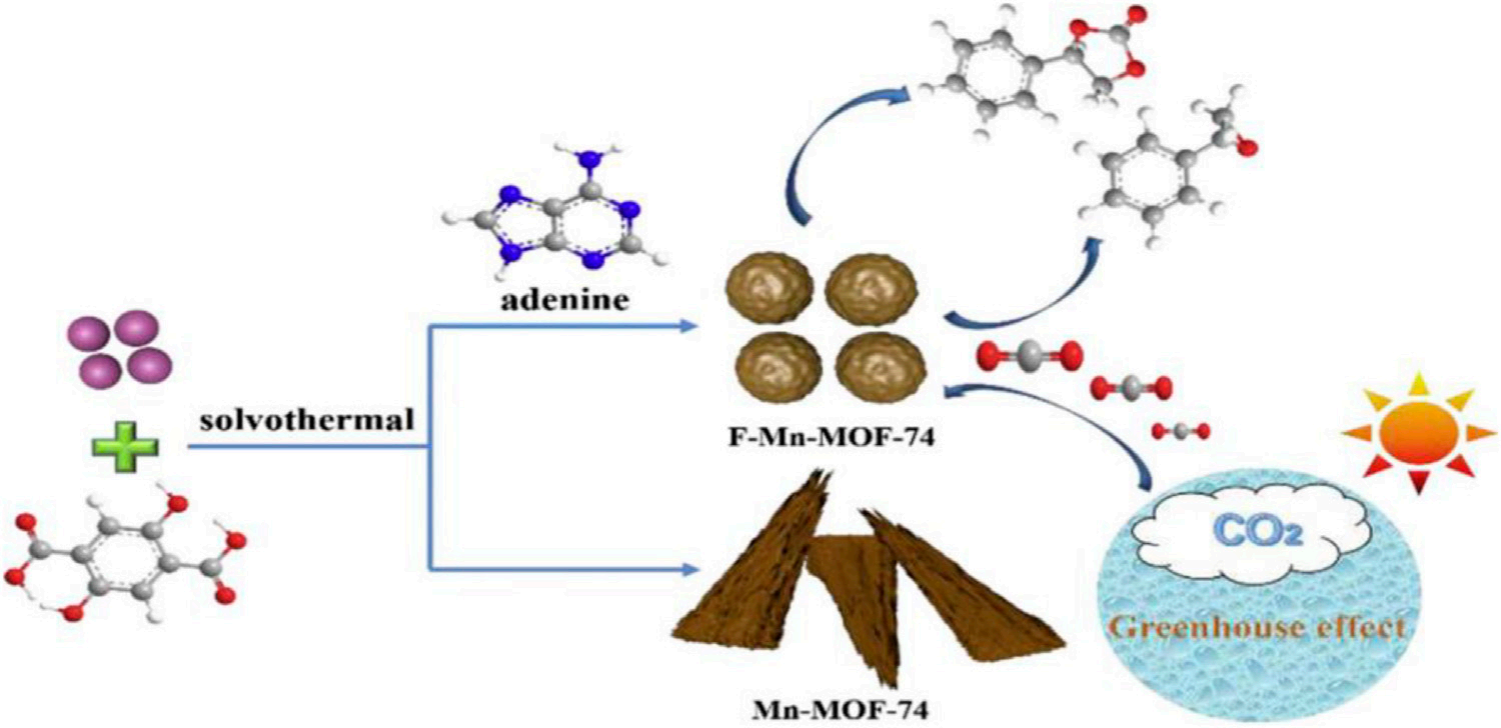
Adenine-assisted synthesis of functionalized F-Mn-MOF-74 heterogeneous catalyst [Reproduced from Ref. (Feng et al., 2020)].

Feng et al. (2020) have functionalized Mn-MOF-74 (**13**) with adenine to synthesize cyclic carbonates. The lone pair of electrons on the nitrogen atom of adenine become a significant ligand which relatively competitive strategy to coordinate with metal ion than the actual ligand 2,5-hydroxyterephthalic acid of MOF-74 (Figure 6).

The formation of cyclic carbonates from esterification reaction between epoxides and carbon dioxide is subject of concern to alleviate the accumulation of carbon dioxide gas in the world. Various homogeneous catalysts like metal-Schiff base complexes, metal oxides and ionic liquids have been developed, but the difficulty with separation and reusability led to the search for alternatives. Lewis acidic-basic active sites rich in MOFs are advantageous in the carbon dioxide cycloaddition reaction. Adenine is a Lewis basic site, and grafting the rich source on the MOF-74 increased the Lewis acid-base catalytic sites’ strength. Nitrogen and oxygen atoms around manganese metal ions serve as Lewis bases, and the adenine improves the Lewis alkalinity. Thus manganese Lewis acidic nature cooperatively enhanced its catalytic activity. The suggested catalytic techniques are helpful in the establishment of sustainable green methodologies.

Aguirre-Díaz et al. (2015) have synthesized α-aminonitriles through Strecker reaction with MOF as a solid catalyst. α-aminonitriles are essential intermediates in the preparation of imidazole and thiadiazole like nitrogen-containing heterocycles. The advantage of MOFs is different chemical compositions and topologies are ideal to act as active sites in the organic transformation. The authors have prepared the incorporation of different metal ions into equivalent positions of the crystalline framework of MOF through a post-synthetic approach termed solid solution MOFs. Initially three isostructural MOFs namely, AlPF-1 [Al(OH) (hfipbb)] (**14**), InPF-11β, [In(O_2_C_2_H_4_)_0.5_ (hfipbb)] (**15**) and GaPF-1, [Ga(OH) (hfipbb)] (**16**) (H_2_hfipbb = 4,4’- (hexafluoroisopropylidene) bis (benzoic acid) were tested for the Strecker reaction. The results attributed to different behaviour catalytically with above said MOFs and forms different products. AlPF-1was employed as the catalyst, resulting in the expected α-aminonitrile. Although the imine formation and the cyanosilylation products formed with **15** and **16**, the target molecule was not achieved. The difference in product formation is ascribed to the different reaction pathways for reactants and the catalyst activation process with each catalyst. The α-aminonitrile can be achieved by combining two pathway reactions in a single entity through the design of solid MOFs. The combination of Ga and In with the general formula [In_x_Ga_1-x_ (O_2_C_2_H_4_)_0.5_ (hfipbb)] demonstrated good catalytic activity to obtain the α-aminonitrile in a one-pot synthesis. The study suggests that modulating different metal ions in the same crystallographic position of the framework structure tends to one-pot multi-component catalytic reactions.

**FIGURE 7:**
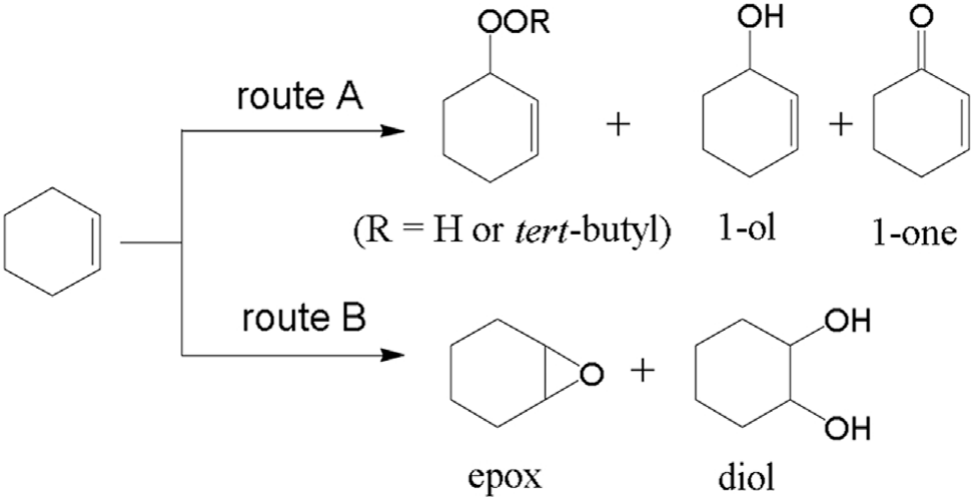
Possible reaction routes of cyclohexene oxidation. [Reproduced from Ref. (Sun and Gao, 2020)].

In another study, Sun et al. (Sun and Gao, 2020) have reported sulfonic-functionalized MIL-101 for the oxidation of cyclohexene. It is a bifunctional catalyst and sulfonic and Cr (III) sites collaboratively enhance the activity. Oxygenated cyclohexene is an essential intermediate for the production of polymers, surfactants, spices and pesticides. Oxidation of cyclohexene at various locations, i.e. at allylic C-H bond and C=C bond, produces multiple products. The limitations with many reported catalysts for selective oxidation of cyclohexene can be overcome using MIL-101-SO_3_H. Grafting the sulfonic group into the MOF can promote the conversion to diol, whereas the chromate group leads to perox formation and1-one. 2-cyclohexen-1,4-dione (dione) product is formed ultimately in route-A with the assistance of the cooperative influence of sulfonic and chromate entities in the structure of MIL-101-SO_3_H (**17**) (Figure 7). Excellent 99% cyclohexene conversion was achieved with **17** but low with MIL-101 alone. The framework provided the bifunctional activity and synergy for selective oxidation of cyclohexene.

## Conclusion

In summary, various novel MOFs have been designed to collate and deal with the practical challenges of industries and R&D institutions in the value-added organic transformations. Heterogeneous catalysis is often described as the potential healer to the technical difficulties of producing organic products/intermediates in conventional synthetic strategies. MOFs offer more incredible promise for improving catalytic efficiency with a good atom economy among various heterogeneous catalysts available for use. MOFs are abundant and inexpensive potential sources to meet the challenges of other heterogeneous catalysts. The inorganic and organic components in the MOFs are reasonably competent to stimulate organic transformations and other applications. MOFs exhibit distinct characteristics that are significantly useful as solid catalysts facilitating conversion of reactants to products with less effort in production and work-up procedures. Most of the MOFs themselves act as catalysts or supports to other catalytic active species. Transition metals are effective catalysts, but lack of heterogeneity tends to look for alternatives. MOFs have become ideal supports, and encapsulation of nano-sized metal particles into the pores of MOFs gives a tremendous advantage for catalysis. In another approach based on the studies, post-synthetic modification of MOFs with catalytically active substances enhance material efficiency. Inherent Lewis acid and base characteristics of MOFs make a creative approach for offering a great scope of applications in heterogeneous catalysis and providing an environmentally sustainable solution. The selection of metal and linker precursors is a prerequisite criterion for acting as a solid catalyst. In most reports, favorable attributes like chiral topologies, unsaturated metal centers, and specific pore apertures have been accomplished in the pre-and post-synthetic approaches. In some cases, bare MOFs have shown little catalytic activity, but anchoring/grafting with other metal ions or functional groups offered substantially improved performance. Overall, this review provides contextual exposure to the MOF chemistry, particularly in heterogeneous catalysis, and is helpful to the researchers to design and construct appropriate MOFs to alleviate the implications associated with the organic transformations.
